# The Epidemiology and Demographics of Legg-Calvé-Perthes' Disease

**DOI:** 10.5402/2011/504393

**Published:** 2011-09-05

**Authors:** Randall T. Loder, Elaine N. Skopelja

**Affiliations:** ^1^Section of Orthopedic Surgery, Riley Hospital for Children, ROC 4250, 705 Riley Hospital Drive, IN, Indianapolis 46202, USA; ^2^Department of Orthopaedic Surgery, Indiana University, Indianapolis, IN 46202, USA; ^3^Ruth Lilly Medical Library, School of Medicine, Indiana University, Indianapolis, IN 46202, USA

## Abstract

The etiology of Legg-Calvé-Perthes' disease (LCPD) is unknown. There are many insights however from epidemiologic/demographic information. A systematic medical literature review regarding LCPD was performed. The incidence ranges from 0.4/100,000 to 29.0/100,000 children <15 years of age. There is significant variability in incidence within racial groups and is frequently higher in lower socioeconomic classes. The typical age at presentation ranges from 4 to 8 years (average 6.5 years), except for children from the Indian subcontinent (average 9.5 years). There is a mild familial component. The children demonstrate impaired growth in height, skeletal age, and birth weight. This impaired growth coincides with an age appropriate reduced somatomedin A activity and decreased levels of IGF. LCPD can be associated with abnormalities in the coagulation cascade, including an increase in factor V Leiden mutation, low levels of protein C and/or S, and decreased antithrombin activity. There is decreased turnover in type I collagen and synthesis of type III collagen, as well as reduced levels of urinary glycosaminoglycans in the active phases of the disorder. Subtle abnormalities in the opposite hip and other minor/major congenital defects are reported. Children with LCPD are active and score abnormally in certain standardized psychological tests.

## 1. Introduction

Legg-Calvé-Perthes' disease (LCPD) is an idiopathic osteonecrosis of the proximal capital femoral epiphysis in children. The epiphysis undergoes collapse, resorption, reossification, and eventual healing. The healed hip may range from an essentially normal contoured femoral head (Stulberg I) to one with incongruous incongruity (Stulberg V). As with any pathologic process, LCPD goes through a course of disease denoted by the Waldenström stages, which are synovitic, avascular, fragmentation (collapse), reossification (healing), and healed (residual). The magnitude of epiphyseal involvement is determined by the Catterall class [1], Salter-Thompson group [2], and/or lateral pillar group [3]. The Catterall class is determined on both anteroposterior and frog-lateral radiographs during the stage of maximum fragmentation, the Salter-Thompson group is determined on the frog-lateral radiograph during the avascular/precollapse stage using the subchondral crescent fracture, and the lateral pillar classification is determined on the anteroposterior radiograph during early fragmentation. 

## 2. Materials and Methods

There are many epidemiologic and demographic findings in LCPD. A systematic review of LCPD was performed. LCPD has been known by at least 22 different names since its first description in the late 19th and early 20th centuries [4]. Since 1963, the official medical subject heading (MESH) used by the National Library of Medicine is Legg-Perthes' disease, but many other names had been previously used. To ensure capture of all the published literature, older terms were also searched as keywords or keyword phrases. Therefore, the terms used to search for LCPD were arthritis deformans juvenilis, Calve-Perthes disease, coxa plana, femoral head necrosis, juvenile chondroepiphysitis, Legg-Calve-Perthes disease, Legg-Perthes disease, Legg's disease, osteochondritis deformans juvenilis, osteochondritis deformans juvenilis coxae, osteochondritis juvenilis, osteochondrosis of capital epiphysis of femur, Perthes disease, and pseudocoxalgia. 

The databases searched were PubMed (http://www.ncbi.nlm.nih.gov/pubmed/), Ovid Medline, EMBASE, WorldCat (books and theses) (http://firstsearch.oclc.org/), and IndexCat (Index Catalogue of the Library of the Surgeon-General's Office) (http://www.indexcat.nlm.nih.gov/). Exclusion criteria were those manuscripts discussing surgery, therapy, rehabilitation, and any foreign language articles without an English abstract. Individual journals were also searched for articles published prior to 1996 that predate electronic Medline indexing, including *Journal of Bone and Joint Surgery (American and British), Clinical Orthopaedics and Related Research*, and *Acta Orthopaedica Scandinavica*. Age groups were limited to those <18 years old. Duplicate citations were removed. The dates for the search were 1880–1961 for *IndexCat*, 1900–2009 for *WorldCat*, 1948–1965 for *OldMedline*, and 1950–February 2010 for *Ovid Medline*. 

This search resulted in 1124 unique citations. These 1124 manuscripts were reviewed to find those that discussed any of the topics regarding etiology, epidemiology, demographics, incidence, prevalence, race, gender, family history, genetics, inheritance, age, bone age, weight (either birth weight or normal weight), height, growth, maturation, other anthropometric characteristics, hormone/endocrine, smoking, coagulation, fibrinolysis, congenital anomalies, collagen, immunoglobulin, opposite hip, behavior/psychology, seasonal variation, and infection. Of these 1124 manuscripts, 144 provided ample information and are the contents of this paper.

## 3. Results

### 3.1. Incidence

The conventional quotation for the incidence of LCPD is the number per 100,000, usually for age < 15 years. The incidence of LCPD ranges widely, from 0.4 in Eastern India (Vellore-Taluk area) to 29.0 (Table 1) in the Faroe Islands (North Atlantic ocean). Significant variability exists within countries, cities, and ethnic groups. Race is classified using the definitions of Eveleth and Tanner: Caucasians, Africans in Africa and of African ancestry, Asiatics (Amerindians, Hispanics, Indonesian-Malays), Indo-Mediterraneans (inhabitants of the Near East, North Africa, and Indian subcontinent), and Australian Aborigines and Pacific Island peoples [23].

### 3.2. Whites

#### 3.2.1. British Isles

**Figure 1:**
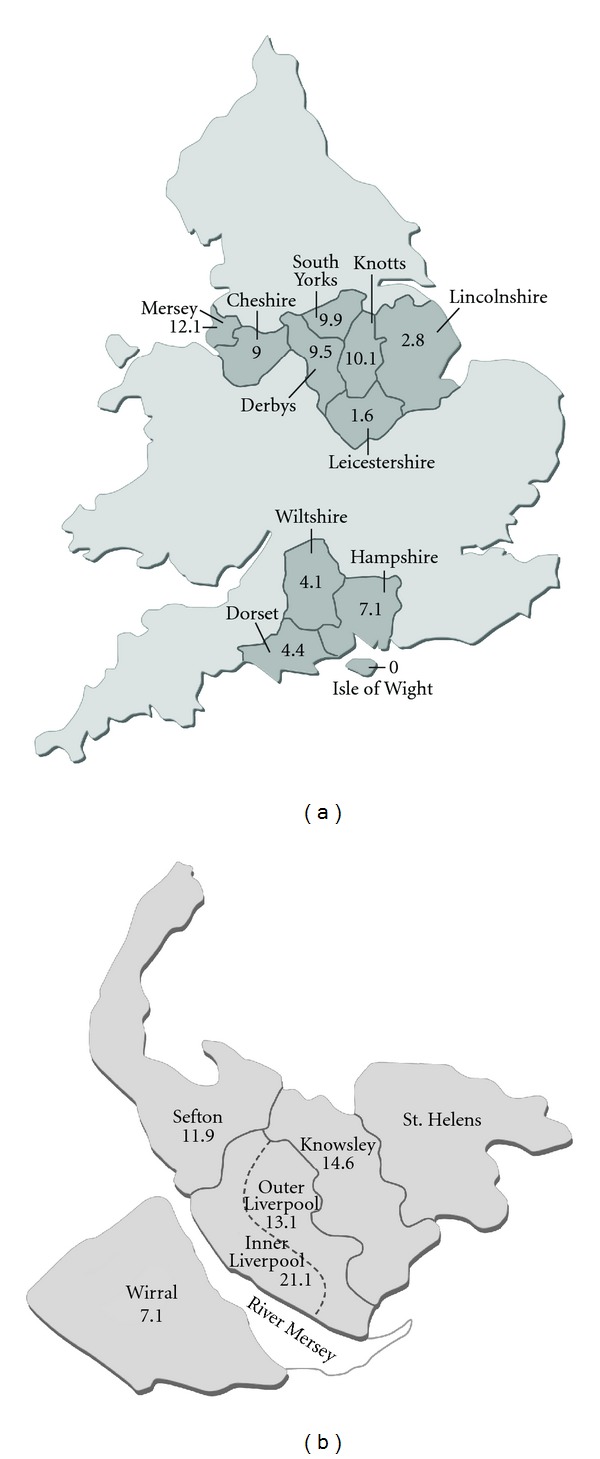
Maps demonstrating various incidences of LCPD in different regions of England. (a) Incidence of LCPD in 1976 per 100,000 children aged 14 years and under in three regions of England. Map of England taken and adapted from the National Policing Improvement Agency, located at http://maps.police.uk/, with permission (Data from [13]). (b) Average yearly incidence of LCPD per 100,000 children aged 14 and under in the Liverpool administrative area. Map of Merseyside area taken and adapted from the National Museums Liverpool located at http://www.liverpoolmuseums.org.uk/maritime/exhibitions/magical/placenames/index.asp, with permission (Data from Hall et al. [21] and Barker et al. [13] (Wirral)).

The incidence ranges from 5.5 in Wessex, England [13], to 15.6 in Liverpool, England [21]. The incidence in 3 different regions of England [13] was 5.5 in the Wessex Health District, 7.6 in the Trent Health District, and 11.1 in the Mersey Health District (including Liverpool) (Figure 1(a)). In Liverpool, the incidence in the inner city was higher (21.1) compared to the surrounding areas (13.1—outer Liverpool, 14.6—Knowsley district, 11.9—Sefton district) [21] (Figure 1(b)) implying that the incidence is lower in less populated or more rural areas. However, in southwest Scotland [20], the incidence was higher in less populated areas (17 to 30) compared to more populated areas (4.5). In Yorkshire, England, which has a substantial rural population, the average incidence was 6.1, with large geographical variations unexplainable by differences between urban and rural populations [15]. The East Riding area of Yorkshire, located on the best agricultural land, had no cases [15]. 

**Figure 2:**
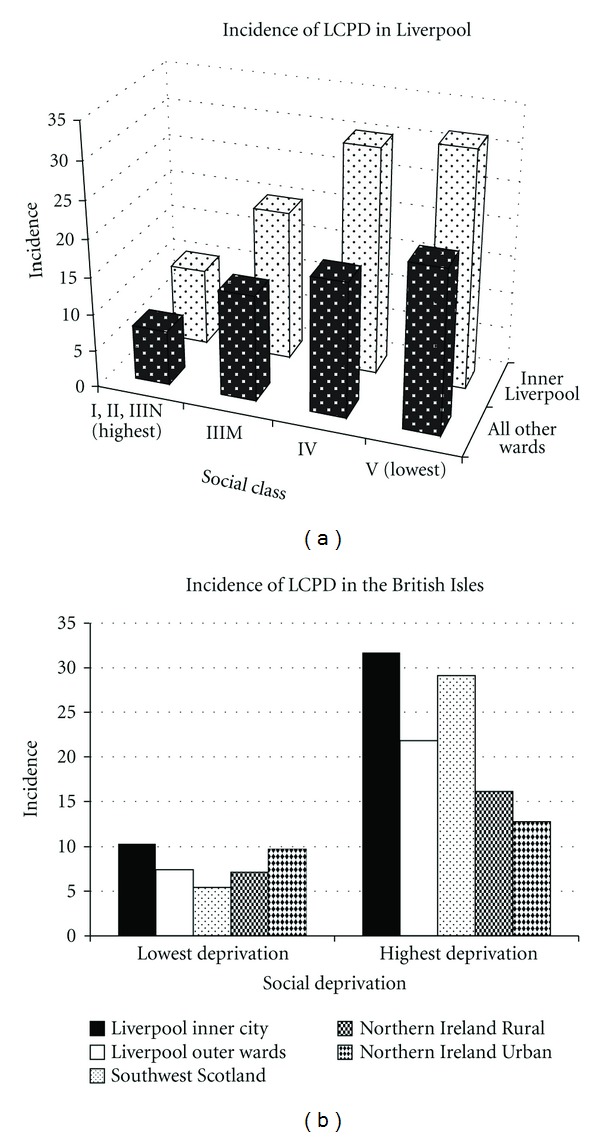
(a) Incidence of LCPD (100,000 children per year ≤14 years of age) by social class in location in Liverpool (Data from Hall et al. [21]). (b) Composite incidence of LCPD (100,000 children per year <14 years of age) by highest and lowest deprivation indices separated by rural and urban locations in the British Isles (Data from Hall et al. [21], Kealey et al. [19], and Pillai et al. [20]).

Many authors have noted differences in incidence by social class and/or inner city/urban/rural location. In the seminal epidemiologic study of 310 children in Edinburgh and Glasgow, Scotland [24], there was a higher than expected proportion of children with LCPD in lower socioeconomic classes; the same was noted in Liverpool [25] (Figure 2(a)). The incidence in the Liverpool inner city within the highest socially deprived area was 31.7 and 10.3 for the lowest; in the outer city the incidence was 21.8 within the highest socially deprived area and 7.4 for the lowest [21] (Figure 2(b)). In Northern Ireland [19], the highest incidence is in the most deprived rural location (16.1), over twice that in the least deprived rural location (7.1). In Southwest Scotland [20], the incidence was 33.6 in the most deprived areas and 7.8 in the least deprived areas; the 33.6 incidence is the highest found to date in any series/publications. However, in Glasgow, there was no association of LCPD incidence and social class [26]. In general, the incidence of LCPD in the British Isles is higher in lower socioeconomic classes and variable regarding rural/urban location (Figure 2(b)). 

#### 3.2.2. Scandinavia

The incidence is 8.5 in Uppsala, Sweden [16], and 9.2 in Norway [17]. Within Norway, similar to the British Isles, there is significant variability; the lowest incidence in the north (5.4) and the highest in the center and west (10.8 and 11.3).

#### 3.2.3. North America

In British Columbia [12], the incidence was 5.10 and, in Massachusetts, [14] 5.7.

#### 3.2.4. Africa

In Eastern Cape, South Africa [6], the incidence in Whites is 10.8; in the urban areas (Port Elizabeth and Uitenhage), it is *∼*2 times greater than in rural areas. This urban-rural dichotomy was noted overall (3.85 versus 1.1) and when separated by race (12.6 versus 6.0 for Whites, 2.2 versus 1.4 for mixed African-White, and 0.7 versus 0.28 for Africans). 

### 3.3. Indo-Malays

In Japan [7], the incidence was 0.90. In Bradford, England, the incidence was 4.6 in Caucasian children and 0.63 in Indo-Malay children [27]. In Korea [9], the incidence was 3.8 and lower in the greater Gwanju metropolitan areas compared to the rural Chonnam province (3.2 versus 4.3). 

### 3.4. Indo-Mediterraneans

#### 3.4.1. India/Sri Lanka

There is a 10-fold variability in incidence in India; 0.4 in the east (Vellore Taluk) [5] to 4.4 in the west (Udupi Taluk). In Sri Lanka (Kurunegala district) the incidence is 3.96 [10], and all 76 children with LCPD were from lower income groups [10].

### 3.5. Africans

True LCPD (excluding sickle cell hemoglobinopathy) is extremely rare in Africans. In Eastern Cape, South Africa [6], the incidence is 0.45 and rises to 1.73 in children of mixed African/Caucasian ancestry. The incidence is 1.8 in Nigeria [8]. In Togo, there were 22 cases of LCPD in 29620 children attending two Togolese hospitals over a 7-year period, indicating the rarity of the disorder. One of 86 children in Massachusetts [14] was African, and two of the 188 children in Connecticut was African [28]. 

### 3.6. Other Demographics (Age, Gender, Laterality, Family History) 

#### 3.6.1. Age, Gender, Laterality, LCPD Severity

The average age is 6.5 years, with a typical age range of 4 to 8 years (Table 2). The average age for Indian children is 9.5 years, for Nigerian children 10.3 years, and for all others 6.3 years. LCPD is more common in boys (81.4%) than girls (18.6%) and mostly unilateral (89.2%). Right and left hip involvement is similar (46.5% and 53.5%). In 1638 hips (Table 3), 112 (6.8%) were Catterall class I, 295 (18.0%) class II, 710 (43.3%) class III, and 521 (31.8%) class IV. In 1671 hips, 236 (14.1%) were lateral pillar group A, 971 (58.1%) B and B/C border, and 464 (27.8%) C. 

#### 3.6.2. Family History/Genetics

A positive family history has been noted by many [7, 24, 27, 28, 37–41]. Quoted percentages are 4.5% [7], 7% [28], and 8% [27]. There are also reports in siblings [42, 43]. The recurrence risk was 2.6% for siblings and offspring in a review of the family histories of 842 English children with LCPD [44], arguing for a multifactorial inheritance pattern. The proportion of the 842 children having a 1st degree relative with LCPD was 1.6%, a 2nd degree relative 0.27%, and a 3rd degree relative 0.27%; all higher than the average English incidence. In South Wales [45], the risk of LCPD in siblings was under 1% and of an affected parent 3%. There are several case reports of LCPD transmitted through several generations [40, 46]. In the Faroe Islands [47], an isolated genetic community, an accumulation of both LCPD and developmental dislocation of the hip was noted in certain families; it remains to be determined if this is genetic, environmental, or both. Others note no significant association with family history [24, 33]. 

There are case reports of LCPD in twins, both monozygotic [48–51] and dizygotic [52], as well as three female 1st degree relatives [53]. These dated studies could not assess for genetic markers, and thus it is unknown if this represents a true genetic pattern or simply the statistical chance of siblings developing the same disease. Wynne-Davies and Gormley [24] described 6 sets of twins with only one of the twins having LCPD.

Several studies show associations with certain HLA types. A positive association was noted with HLA-A_1_ [54, 55] and HLA-A_9_, HLA-A_10_, and HLA-B_27_ [56]. A protective effect was seen with HLA-A_2_ and HLA-Cw3; the incidence of LCPD was less in those types [57]. Two studies found no differences in HLA types [58, 59]. There is no apparent association between ABO and Rh blood groups [60]. 

### 3.7. Perinatal Factors (Parental Age, Birth Order/Presentation, Birth Weight)

Both parents of children with LCPD were older than the normal population (31.7 versus 28.8 years for the fathers, 28.9 versus 26.9 for the mothers) in one study [24], with no differences noted in parental age by others [28, 33, 61]. 

LCPD was more frequent in the 3rd born or older children [24] in one study, while others noted no differences [33]. Children with LCPD are more commonly born breech—10.7% compared to 2–4% in the normal population [24]. A lower birth weight was noted in children with LCPD [62, 63]; 7.1 lbs in 70 children with LCPD and 7.8 lbs in 70 control children without LCPD [62]. In 5 sets of twins, the smaller twin at birth developed the LCPD [63]; the average discordance in birth weight was 13.4% (range 7.1 to 23.5%). Others note no differences in birth weight [24, 33, 61]. 

### 3.8. Impaired Growth, Anthropometric Differences, and Skeletal Maturation

#### 3.8.1. Impaired Growth and Anthropometric Differences

Height retardation was noted in 185 Ohio children with LCPD [64], even when accounting for parental height; body weight was average or above average. In Scottish children, a greater proportion of LCPD children have diminished height (<10th percentile) with no differences for weight [24]. In 76 Sri Lanka children with LCPD, 46% were below average height at presentation [10]. In 109 Japanese children with LCPD, 97 (89%) were below the mean in height [65]. Children with LCPD are shorter at birth and remained so during the phases of LCPD and adulthood [66, 67]; boys were 4.4 cm shorter and girls 2.5 cm shorter than their norms [66]. No height or weight differences were found in Irish [68] and Jewish children with LCPD [69].

Skeletal growth is progressively impaired in a caudal direction. Rostral sparing is documented by normal head growth [70] with increasing growth retardation in a caudal direction: biacromial width was less reduced than standing height; forearm and hand showed more impaired growth than the upper arm; the feet showed more impaired growth than the leg. This impaired growth most severely affects the feet [24, 71]. Growth retardation in LCPD children from rural India [72] is identical to English children. 

#### 3.8.2. Skeletal Maturation

**Figure 3:**
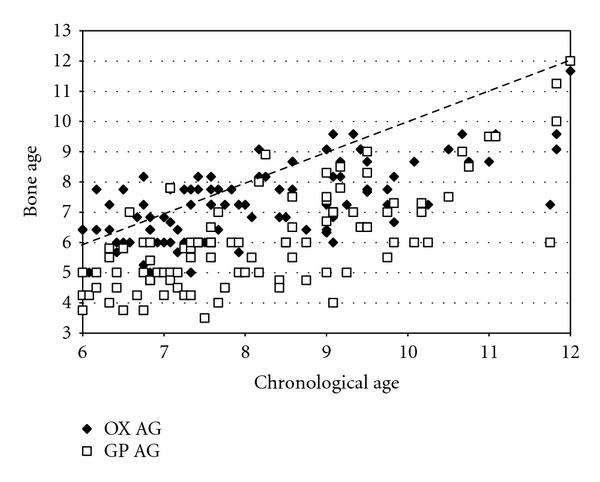
Chronologic age as a function of bone age in 100 children with LCPD. Both the hand-wrist bone of Greulich and Pyle (GP AG) [73] as well as the Oxford pelvic bone age (OX AG) [74] are shown (Data from the study of Loder et al. [75]).

Aside from one study in Jewish children with LCPD [69], all others note delayed bone age in LCPD. In 182 children with LCPD, many were <3rd percentile bone age, which was more common in boys than girls [76]. In 125 of 140 (89%) children with LCPD [28] bone age was delayed. Bone age was at least 3 months less in 83% of children [67]. This is seen [75] with both the Greulich-Pyle hand-wrist assessment [73] and the Oxford pelvis method [74] of determining bone age (Figure 3). The average chronologic age for both boys and girls was 8.2 years; for boys, the average hand-wrist bone age was 7.4 years and average pelvic bone age 5.9 years, and, for girls, the average hand-wrist bone age was 6.9 years and average pelvic bone 7.0 years [75]. Carpal maturation was delayed in 125 children with LCPD; the most severe delay was at 3 to 5 years of age [77]. In a study of 27 girls with LCPD at the time of diagnosis [78], bone age (Tanner Whitehouse 2 method) was delayed an average of 1.4 years for the radius/ulna and 1.9 years for the carpals. A greater delay in bone age is associated with more severe LCPD [79]. Children with transient synovitis show minimal delay in bone age compared to those with LCPD (7 months versus 23 months) [80]. 

In non-Caucasians, bone age was delayed 31.8 months in 17 of 25 Formosan (68%) children with LCPD [33]. In Hong Kong, all Chinese children with LCPD had a bone age lower than the mean [11]. In Korean children, bone age was delayed 10.4 months in boys and 4.6 months in girls [9]. In 21 Japanese LCPD children, delayed bone age was noted in all [65]. In 76 Sri Lankan children, 78% demonstrated skeletal retardation [10]. In Mexican children (Hispanic-Amerindian), bone age was delayed 28 months in children with LCPD between the ages of 6–10 years [81]. 

Skeletal standstill (no increase in bone age with increasing chronologic age) occurs in LCPD [75, 76] and resolves after the LCPD has healed [80].

### 3.9. Endocrine Dysfunction

Postnatal skeletal development is regulated by growth hormone, whose effects are partly mediated by somatomedins. Somatomedins stimulate cartilage activity resulting in cell proliferation and hypertrophy. In Japan, the incidence of LCPD was 70 in growth-hormone-deficient children [82] compared to 0.9 in the normal population [7]. Serum growth hormone response to insulin-induced hypoglycemia is reduced in boys with LCPD compared to those with constitutional short stature [83]. The primary somatomedin responsible for postnatal skeletal maturation is somatomedin C insulin-like growth factor (IGF-1). Somatomedin deficiency may result in impaired skeletal maturation, a well-known phenomenon in LCPD. Somatomedin A [84] and C [65] deficiency has been noted LCPD. Somatomedin activity normally increases with age in growing children, but this does not occur in children with LCPD [85, 86]. Plasma levels of IGF-1 were reduced the first 2 years after the diagnosis of LCPD [87], but with normal levels of IGF-binding protein [88]. Low levels of IGF-1 were confirmed by Crofton et al. [89], who also noted abnormal collagen turnover in the acute stages of LCPD. In plasma, nearly all the IGF-1 is bound to specific binding proteins, which for IGF-1 is the IGF-binding protein 3 (IGFBP3). Decreased levels of IGFBP in children with LCPD have been seen but with normal levels of IGF-1 [90]. No abnormalities in IGF-1 or IGFBP concentrations have been encountered by others [68, 83, 91, 92] in children with LCPD. 

Early studies [93, 94] noted an association with hypothyroidism and LCPD but not seen in more recent studies [68, 83, 95–98]. No abnormalities in adrenal function (cortisol) [68, 83] or cholesterol [96] have been noted. 

### 3.10. Smoking, Hypofibrinolysis, and LCPD

Passive smoke exposure during pregnancy has been correlated with LCPD. This was first noted in Massachusetts [62]; maternal smoking while pregnant was present in 63% of LCPD and 43% of control cases. This was confirmed in Sweden [99]; maternal smoking during pregnancy increased the odds of developing LCPD in the child by 1.44 if the mother smoked <10 cigarettes per day, and by 2.1 when ≥10 cigarettes per day. It was also noted that children with a birth weight <1500 gms had a 2.4 times increased risk of developing LCPD. 

**Figure 4:**
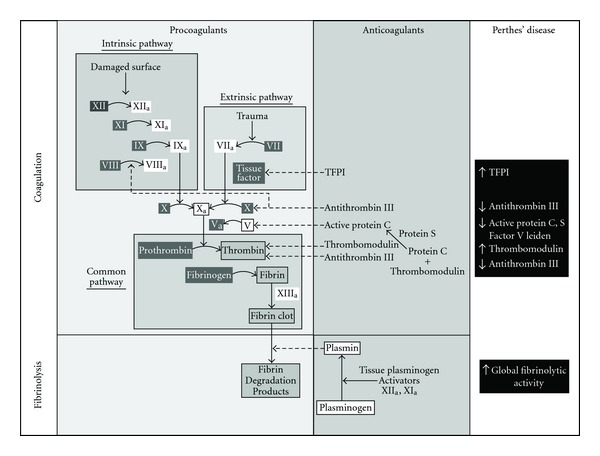
The coagulation and fibrinolytic cascade as it relates to children with LCPD. The abnormalities in this cascade in children with LCPD are shown in the far right column. TFPI: tissue factor pathway inhibitor.

An increase in LCPD in children exposed to passive smoke after birth has also been noted. In children with LCPD [100], 63.9% had at least one smoker living in the child's household with a mean of 1.03 smoker years per year of life exposure to smoke; in control children, 39.6% had at least one smoker living in the child's house with a mean of 0.48 smoker years per year of life exposure to smoke. No association was noted between lower income and LCPD. This association with passive smoke exposure was corroborated in Spain [101], where 79% of LCPD children were passive smokers compared to 43% of controls. The odds ratio for a child, after controlling for age and gender, of developing LCPD when exposed to passive smoke was 5.3 (95% CI 2.9–9.7). There were no associations between passive smoking and age of child, Catterall class, or final Stulberg result. In another study of 39 children with LCPD [102], 24 had exposure to second hand smoke, some even in utero (17 of the 24). Of the children with LCPD and smoke exposure, 48% had low stimulated tissue plasminogen activator activity, compared to only 7% of the children without smoke exposure. In Georgia [103], children exposed to passive smoke were 5.6 (95% CI 2.0–12.0) times more likely to develop LCPD than those not exposed. This was strongly associated with a polymorphism in the *β*-fibrinogen gene G-455-A, which results in increased fibrinogen levels, thus leads to thrombotic/coagulation abnormalities in children with LCPD (Figure 4).

Factor V Leiden mutation discovered in Leiden, Netherlands [104], results in production of factor V that cannot be inactivated by activated protein C. This leads to a persistence of circulating activated factor V with continued activation of the coagulation cascade and a hypercoagulable state. Families with LCPD and factor V Leiden mutations have been described [105, 106]. In nonfamilial LCPD, a factor V Leiden mutation has been noted by many; 12.5% [107] and 10.6% in children with LCPD [108] in studies without controls. In studies with controls, these values are 30% in LCPD and 1.87% in controls [109], 11% in LCPD and 4% in controls [110], 9% in LCPD and 5% in controls [111], and 4.9% in LCPD and 0.7% in controls [112]. Children with the most severe LCPD (Catterall IV) were homozygous for factor V Leiden mutation [108]. High levels of anticardiolipin antibodies (26% versus 11%) have also been noted [110]. The OR of developing LCPD with factor V Leiden mutation in two studies are 22.5 [109] and 3.3 [113]; the OR of developing LCPD with ≥ abnormalities in factor V or anticardiolipin antibody is 3.29 [110].

Other coagulation abnormalities exist in LCPD. Thrombophilia and hypofibrinolysis were noted in 8 children [114] in 1994. A subsequent investigation noted that 75% of 44 children with LCPD had coagulation abnormalities [115]; thrombophilia (a deficiency in antithrombotic factor C or S, with an increased tendency towards thrombosis) in 23 children; increased lipoprotien(a) (a thrombogenic lipoprotein associated with osteonecrosis in adults) in 7 children; hypofibrinolysis (reduced ability to lyse clots) in 3 children. In another study, only 14 of 64 children (22.5%) with LCPD had entirely normal coagulation measures [107] with resistance to activated protein C the most common abnormality (23 of 64). A 3.8 times increased risk for LCPD with low levels of protein C has been found [111]. Protein C activity is also lower in LCPD [116, 117]. Both protein C and antithrombin activities were lower in LCPD than controls [117]; a family history of hereditary thrombophilia was higher in LCPD than controls. LCPD was increased 2.8 times with protein S deficiency and 7.5 times with elevated factor VIII levels [113]. Others note no coagulation abnormalities in LCPD [116, 118–127].

Another fact supporting a hypercoagulable state in LCPD is tissue factor pathway inhibitor (TFPI). TFPI is an important natural anticoagulant molecule that downregulates the tissue factor dependent coagulation pathway. A deficiency leads to a prothrombotic state, and over expression may be a protective mechanism against ongoing local microvascular events. TFPI concentrations in children with LCPD were significantly higher (56.8 ng/mL) compared to controls (37.3 ng/mL) [128]. This is interpreted as a physiologic response to a hypercoagulable state; an increased TFPI is natural anticoagulation. Increased blood viscosity in LCPD is reported [129]; thus vascular occlusion may simply be due to fluid mechanic properties [129].

After thrombosis, the body attempts to lyse the clot. Fibrinolysis is mediated in part by thrombomodulin, an endothelial cell membrane-associated glycoprotein which functions in activation of the anticoagulant systems. In LCPD, thrombomodulin and global fibrinolytic activity are elevated [130]. This is interpreted as a compensatory reaction to the thrombosis in LCPD.

### 3.11. Other Associations with LCPD

#### 3.11.1. Collagen Metabolism/Genetics and Bone Turnover

**Figure 5:**
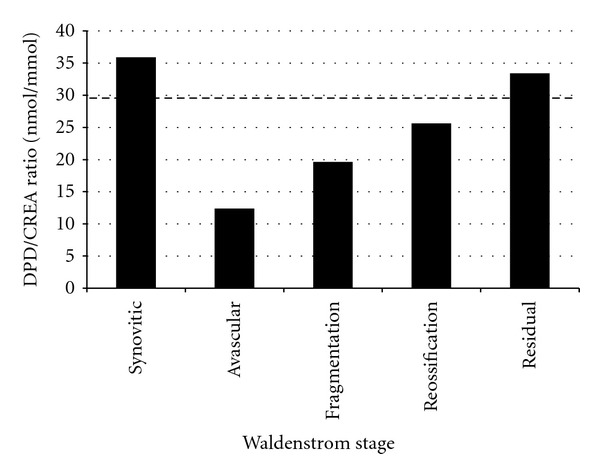
Urinary DPD/CREA (urinary deoxypyridinoline/creatinine ratio) in children with LCPD in different Waldenström stages. The control level is denoted by the hatched line. DPD is a degradation product of type I collagen; a decrease in its urinary excretion indicates a decrease in bone turnover. Thus, there is decreased bone turnover in children with LCPD during the avascular and fragmentation phases of the disease (Data from Westhoff et al. [131]).

Type I collagen is found almost exclusively in bone and calcifying tissues. Markers of type I collagen degradation are urinary deoxypyridinoline (DPD) and type I collagen telopeptide (ICTP). ICTP is higher in children with LCPD compared to controls [89], indicating an increase in type I collagen degradation. The median urinary DPD/creatinine ratio in children with LCPD is reduced during the fragmentation stage and returns to normal (if not slightly higher) in the healed stage [131] (Figure 5). The DPD/creatinine decrease is greater with more severe LCPD (egg lateral pillar C > lateral pillar B). These findings support a systemic etiology in LCPD. 

A marker of type III collagen synthesis is the procollagen type III N-terminal propeptide (P3NP). Type III collagen synthesis is reduced at diagnosis in children with LCPD as demonstrated by very low levels of P3NP. However, there were no controls, and the differences in children with LCPD compared to otherwise normal children in the same geographic/ethnic/socioeconomic situation are not known. 

A recurrent mutation in type II collagen (cartilage collagen) in a Japanese family with LCPD [38] has been noted. This mutation amino acid change (p.G1170S) perturbs the Gly-X-Y triple-helix of type II collagen. Similar findings were noted in a Chinese family where a p.Gly1170S mutation of COL2A1 resulted in premature hip osteoarthritis, avascular necrosis of the femoral head, or LCPD, depending upon the age at onset [39]. In a cohort of nonfamilial children with LCPD, no mutations in the COL2A1 gene were found [124].

#### 3.11.2. Articular Cartilage Markers

Glycosaminoglycans (GAGs) are chains of repetitive disaccharide units linked with proteins in the cartilaginous extracellular matrix to form proteoglycans. Upon cartilage degradation, GAGs are eliminated by the kidneys. Elevated urinary GAG levels indicate increased articular cartilage degradation. Decreased levels of urinary GAGs in children with LCPD compared to normal children or those with transient synovitis have been noted [132]. This can be interpreted as either increased preservation of the GAGs within the hip or a decrease in the quantity of synovial fluid. Increased levels of proteoglycan fragments and stromelysin in the synovial fluid of children with LCPD have been noted, consistent with a synovitis [133].

#### 3.11.3. The Opposite Hip in Unilateral LCPD

In a review of the radiographs of 153 children with unilateral LCPD [134], 48.4% demonstrated irregularity of the epiphyseal surface, flattening, or dimpling of the opposite “normal hip.” In most instances (37%), they were present in the initial radiograph. Similar changes were noted in only 10.4% of a control group of 153 age and gender-matched children using intravenous urograms. This was interpreted as the capital femoral epiphysis in the young child being very vulnerable to stress; the minimal contour irregularities in the “normal hip” represent one end of the spectrum and frank LCPD, the other as the stress response of the capital femoral epiphysis. Another study confirmed that the “unaffected” hip in LCPD demonstrates anterior and lateral flattening perhaps indicating a constitutional abnormality [135]. In a third study, 15% of the opposite “normal” hips demonstrated physeal changes, especially decalcification below the physis [136]. The initial radiographs of 125 Japanese children with unilateral LCPD demonstrate delayed ossification of the opposite epiphysis as seen by diminished epiphyseal height [137]. 

#### 3.11.4. Behavioral/Psychological Issues

Children with LCPD are extremely busy and active. An early study (PhD thesis) discovered that children with LCPD demonstrated a motor-expressive personality, an active approach to life and had higher psychosomatic and visceral complaints [138]. A later study [139] reviewed the behavioral characteristics of 24 children with LCPD; 33% of children with LCPD had abnormally high scores in standard psychological child behavioral questionnaires for profiles associated with attention deficit hyperactivity disorder, greater than the 3–5% of age matched children. Certain epidemiologic characteristics of LCPD (gender, socioeconomic status, geographic location, and associated congenital anomalies) are also similar characteristics of attention deficit hyperactivity disorder. These findings were confirmed in a recent study of 19 children with LCPD [92]; 8 of 12 school-aged children had negative scores in neuropsychological tests and 5 of the 8 had learning difficulties at school. 

#### 3.11.5. Miscellaneous Findings

An increase in both major and minor congenital defects in children with LCPD is known [61]. These include anomalies of the genitourinary tract and inguinal region [140] and spina bifida occulta [10, 141, 142]. Sacral inclination, decreased lumbar lordosis, and an overall more negative spinal balance with vertebral end plate anomalies have been recently described in the spine of LCPD patients [143]. 

Low blood manganese levels were noted in children with LCPD in Liverpool [144], but refuted by others [145]. An increase in IgG and IgM, but not IgA serum immunoglobulin levels in LCPD, are described [146], suggesting that immunological mechanisms may mediate certain changes in LCPD. Rubella antibody titers are higher in both mothers and affected children with LCPD [147]. 

## 4. Conclusion and Unifying Possibilities

Can these epidemiologic and demographic findings be unified? There clearly is disharmony between cartilage and bone and growth in LCPD as evidenced by progressive caudal growth impairment and delays in skeletal maturation, both involving the wrist and the pelvis. The insult on skeletal maturation appears to occur early in life, perhaps even prenatally, since there is an increased frequency of minor congenital malformations in children with LCPD. These delays in maturation (both anthropometric and skeletal age) can be due to a combination of familial and environmental circumstances (lower socioeconomic class with malnutrition [148], underlying genetic/collagen defects, or some other unknown entity). The delay in skeletal ossification results in a weaker skeleton that is more susceptible to trauma. A highly active child incurs more skeletal injuries; this microtrauma in a biologically susceptible weaker skeleton creates microfractures in the proximal femoral epiphysis and metaphysis. A hypercoagulable state, due to underlying abnormalities in the clotting mechanisms and/or exposure to passive smoke, results in increased thrombosis in the proximal femur after microfractures with subsequent necrosis of the capital femoral epiphysis and the development of LCPD. 

##  Conflict of Interests

The author's otherwise have no financial interests with any other organizations or bodies.

##  Disclosure

As a systematic literature review, Institutional Review Board approval is not applicable. This was the 2nd of three presentations on the epidemiology and demographics of Pediatric Hip Disorders given at the AO North American Symposium on Surgical Preservation of the Hip, Squaw Valley, California, January 2009.

## Figures and Tables

**Table 1:** Incidence of Legg-Calvé-Perthes' disease*.

Study	Year	City, country	Region	Ethnicity	No Pts	Incid
Joseph et al. [5]	1988	Vellore, India	Asia	Indo-Med (Indian)	4	0.4
Purry [6]	1982	Eastern Cape, South Africa	Africa	Black	6	0.45
Kim et al. [7]	2006	Japan	Asia	Indo-Malay (Japanese)	711	0.9
Purry [6]	1982	Eastern Cape, South Africa	Africa	Mixed	11	1.73
Ebong [8]	1977	Nigeria	Africa	Black	10	1.8
Rowe et al. [9]	2005	Chonnam, Korea	Asia	Indo-Malay (Korean)	84	3.8
Joseph et al. [5]	1988	Udupi, India	Asia	Indo-Med (Indian)	138	4.4
Wijesekera [10]	1984	Kurunegala, Sri Lanka (Ceylon)	Asia	Indo-Med (Indian)	76	3.96
Thompson and Leong [11]	1978	Hong Kong	Asia	Indo-Malay (Chinese)	32	4.5
Gray et al. [12]	1972	British Columbia, Canada	North America	White	379	5.1
Barker et al. [13]	1978	Wessex, England	British Isles	White	34	5.5
Molloy and MacMahon [14]	1966	Massachusetts	North America	White^†^	86	5.7
Hall and Barker [15]	1989	Yorkshire, England	British Isles	White	101	6.1
Barker et al. [13]	1978	Trent, England	British Isles	White	78	7.6
Moberg and Rehnberg [16]	1964	Zealand, Denmark	Scandinavia	White	NA	8.0
Moberg and Rehnberg [16]	1992	Uppsala, Sweden	Scandinavia	White	51	8.5
Moberg and Rehnberg [16]	1964	Jutland, Denmark	Scandinavia	White	NA	9.0
Wiig et al. [17]	2006	Norway	Scandinavia	White	425	9.2
Purry [6]	1982	Easter Cape, South Africa	Africa	White	38	10.8
Margetts et al. [18]	2001	Liverpool, England	British Isles	White	122	11.1
Barker et al. [13]	1978	Mersey, England	British Isles	White	68	11.1
Kealey et al. [19]	2000	Northern Ireland	British Isles	White	313	11.6
Pillai et al. [20]	2005	Dumfries, Scotland	British Isles	White	40	15.4
Hall et al. [21]	1983	Liverpool, England	British Isles	White	157	15.6
Niclasen [22]	1974	Faroe Islands, Denmark	Scandinavia	White	43	29.0

*(per 100,000 children <15 yrs old).

^†^one of the 86 children was African.

NA: not available.

**Table 2:** Demographics of 4166 children with Legg-Calvé-Perthes' disease.

Study	Year	City, country	Region	Race	No Pts	Age (yrs)	M (%)	F (%)	Unil (%)	Bil (%)	R (%)	L (%)
Rosenfeld et al. [29]	2007	Dallas, TX	North America		172	4.6			156 (90.7)	16 (9.3)		
Catterall [1]	1971	London, England	British Isles	White	121	4.66	96 (79.3)	25 (20.7)	109 (90.1)	12 (9.9)		
Gray et al. [12]	1972	British Columbia, Canada	North America	White	379	5	322 (85.0)	57 (15.0)	317 (84.1)	60 (15.9)	150 (47.3)	167 (52.7)
Kealey et al. [19]	2000	Northern Ireland	British Isles	White	313	5.7	256 (81.8)	57 (18.2)	264 (84.3)	49 (15.7)		
Moberg and Rehnberg [16]	1992	Uppsala, Sweden	Scandinavia	White	51	5.75	38 (74.5)	13 (25.5)	45 (88.2)	6 (11.8)		
Hall et al. [21]	1983	Liverpool, England	British Isles	White	157	5.8	133 (84.7)	24 (15.3)				
Wiig et al. [30]	2008	Norway	Scandinavia	White	425	5.8	324 (76.2)	101 (23.8)	370 (87.1)	55 (12.9)	167 (45.4)	201 (54.6)
Fulford et al. [31]	1993	Edinburgh, Scotland	British Isles	White	94	5.8	85 (90)	9 (10)	89 (95)	5 (5)		
Fisher [28]	1972	Connecticut	North America	White	203	6	153 (81.4)	35 (18.6)	163 (86.7)	25 (913.3)	83 (50.9)	80 (49.1)
Rowe et al. [9]	2005	Chonnam province, Korea	Asia	Indo-Malay (Korean)	84	6			79 (94)	5 (6)	46 (52)	43 (48)
Pillai et al. [20]	2005	Dumfries, Scotland	British Isles	White	40	6.5	31 (78)	9 (22)				
Guille et al. [32]	1998	Wilmington, DE	North America		575	6.9	470 (81.7)	105 (18.3)	497 (86.4)	78 (13.6)		
Wang et al. [33]	1990	Taipei, Taiwan	Asia	Indo-Malay (Chinese)	57	7	47 (83)	10 (17)	52 (91)	5 (9)	21 (40)	31 (60)
Kim et al. [7]	2006	All Japan	Asia	Indo-Malay (Japanese)	711	7.08	606 (86.4)	95 (13.6)	656 (92.3)	55 (7.7)	281 (43.0)	327 (57.0)
Petrie and Bitenic [34]	1971	Montreal, Quebec	North America	White	60	7.75	49 (82)	11 (18)	41 (84)	8 (16)		
Herring et al. [35]	2004	All USA	North America		345	8	271 (80.4)	66 (19.6)	317 (91.9)	28 (8.1)	162 (47.0)	183 (53.0)
Joseph et al. [5]	1988	Karnataka and Vellore, India	Asia	Indo-Med (Indian)	138	9.4	97 (70.3)	41 (29.7)				
Chacko et al. [36]	1986	Karnataka, India	Asia	Indo-Med (Indian)	165	9.6	119 (72.1)	46 (27.9)	155 (93.9)	10 (6.1)	92 (54.9)	63 (40.6)
Wijesekera [10]	1984	Kurunegala, Sri Lanka (Ceylon)	Asia	Indo-Med (Sri Lankan)	76	9.3	52 (68)	24 (32)	74 (97)	2 (3)	40 (53)	34 (47)

			All			6.5	4237 (81.4)	969 (18.6)	4039 (89.2)	491 (10.8)	1227 (46.5)	1410 (53.5)
Averages			Non-Indian			6.3						
			Indian			9.5						

**Table 3:** Severity of epiphyseal involvement in Legg-Calvé-Perthes' disease.

Study	Year	Location	Catterall class				Lateral pillar group				
			I	II	III	IV	A	B	BC	B + BC	C
Rosenfeld et al. [29]	2007	Dallas, TX					7	108	30	138	43
Catterall [1]	1971	London, England	31	31	22	13					
Guille et al. [32]	1998	Wilmington, DE	37	120	228	233	155	204		204	204
Wang et al. [33]	1990	Taipei, Taiwan	0	7	19	21					
Kim et al. [7]	2006	Japan	30	103	352	210	68	350		350	157
Herring et al. [35]	2004	USA					6	218	61	279	60
Wijesekera [10]	1984	Kurunegala, Sri Lanka	13	21	33	11					
Chacko et al. [36]	1986	Karnataka, India	1	13	56	33					

Total			112	295	710	521	236	880	91	971	464

Percentage			6.8	18.0	43.3	31.8	14.1	52.7	5.4	58.1	27.8
